# *β*‐GPA treatment leads to elevated basal metabolic rate and enhanced hypoxic exercise tolerance in mice

**DOI:** 10.14814/phy2.13192

**Published:** 2017-03-14

**Authors:** Trenton T. Ross, Jeffrey D. Overton, Katelyn F. Houmard, Stephen T. Kinsey

**Affiliations:** ^1^ Department of Biology and Marine BiologyUniversity of North Carolina WilmingtonWilmingtonNorth Carolina

**Keywords:** Basal metabolic rate, exercise, *β*‐GPA

## Abstract

Treatments that increase basal metabolic rate (BMR) and enhance exercise capacity may be useful therapeutic approaches for treating conditions such as type 2 diabetes, obesity, and associated circulatory problems. *β*‐guanidinopropionic acid (*β*‐GPA) supplementation decreases high‐energy phosphate concentrations, such as ATP and phosphocreatine (PCr) resulting in an energetic challenge that is similar to both exercise programs and hypoxic conditions. In this study, we administered *β*‐GPA to mice for 2 or 6 weeks, and investigated the effect on muscle energetic status, body and muscle mass, muscle capillarity, BMR, and normoxic and hypoxic exercise tolerance (NET and HET, respectively). Relative [PCr] and PCr/ATP ratios significantly decreased during both treatment times in the *β*‐GPA fed mice compared to control mice. Body mass, muscle mass, and muscle fiber size significantly decreased after *β*‐GPA treatment, whereas muscle capillarity and BMR were significantly increased in *β*‐GPA fed mice. NET significantly decreased in the 2‐week treatment, but was not significantly different in the 6‐week treatment. HET significantly decreased in 2‐week treatment, but in contrast to NET, significantly increased in the 6‐week‐treated mice compared to control mice. We conclude that *β*‐GPA induces a cellular energetic response in skeletal muscle similar to that of chronic environmental hypoxia, and this energetic perturbation leads to elevated BMR and increased hypoxic exercise capacity in the absence of hypoxic acclimation.

## Introduction

Acute exercise induces an energetic challenge in skeletal muscle by increasing the rate of ATP consumption, which is manifested as a transient reduction in the phosphocreatine (PCr) to ATP ratio during the exercise period, followed by a relatively rapid return to resting levels postexercise (Kushmerick and Conley 2002). During exercise, the creatine kinase (CK) reaction catalyzes the net transfer of a phosphoryl group from PCr to ADP, forming ATP and creatine, whereas the reaction operates in the net reverse direction during recovery from exercise when ATP demand returns to resting levels (Ellington 2001). Unlike the transient effects on energy state that occur during short‐term exercise, chronic endurance exercise training can lead to a shift toward more aerobic, slow‐twitch muscle fiber types, which have a persistently lower resting PCr/ATP ratio (Bernus et al. 1993; Hoff et al. 2013) and higher resting metabolic rate than fast‐twitch fiber types (Blei et al. 1997).

Hypoxia can also lead to a reduced PCr/ATP ratio, but unlike exercise, this is not due to an increased rate of ATP consumption. We have previously shown that acclimation to environmental hypoxia in mice leads to a decrease in the skeletal muscle PCr/ATP ratio and an increase in the AMP/ATP ratio, and this is associated with enhanced hypoxic exercise tolerance (Overton et al. 2009). Similarly, Hochachka et al. (1996) showed that cardiac muscle from humans indigenous to high altitude have a reduced PCr/ATP ratio that appears to be part of a suite of adaptions that promotes enhanced endurance under hypoxic conditions. Likewise, a reduced PCr/ATP ratio was found in climbers after an extended excursion to high altitude (Holloway et al. 2011). Thus, a resting muscle energetic profile that is more akin to the exercising state appears to be an acclimation or adaptive response to hypoxia that leads to enhanced hypoxic exercise tolerance.

*β*‐guanidinopropionic acid (*β*‐GPA) is a creatine (Cr) analog that inhibits creatine uptake by the cell. *β*‐GPA can be slowly phosphorylated by CK to form P‐GPA, however, *β*‐GPA and P‐GPA are inefficient substrates for the CK reaction, yielding a reaction rate that is <1% of that for creatine and PCr (Fitch et al. 1978; Chevli and Fitch 1979). *β*‐GPA supplementation leads to a reduction in the PCr/ATP ratio (and an increase in AMP/ATP) in skeletal muscle (Oudman et al. 2013) causing the activation of the energy‐sensing enzyme, AMP‐activated protein kinase (AMPK) (Bergeron et al. 2001; Williams et al. 2009; Baumgarner et al. 2015). Since both endurance training and acclimation to environmental hypoxia lead to a reduced PCr/ATP in skeletal muscle, we hypothesized that reducing the PCr/ATP ratio using *β*‐GPA would increase BMR and hypoxic exercise tolerance. Although *β*‐GPA has been used as a dietary supplement for several decades, to our knowledge no research has been conducted to date examining the effect of *β*‐GPA supplementation on whole body BMR and hypoxic endurance.

## Materials and Methods

### Animal care and maintenance

C57BL/6J mice (*Mus musculus*) were purchased from The Jackson Laboratory (Bar Harbor, Maine) and were maintained at the University of North Carolina Wilmington (UNCW). Mice were maintained in polycarbonate cages under laminar flow hoods at 22–24°C, provided with Agway 3000 mouse food ad libitum, and maintained with a 12 h light/dark cycle. Offspring were removed from mating pairs and separated by sex at 3 weeks of age, with no more than three mice per cage. At 10 weeks of age, mice were randomly assigned to one of three experimental groups. Control mice received powdered mouse food with no added supplements for 6 weeks. The 2‐week *β*‐GPA treatment received powdered mouse food with no supplements for 4 weeks, and then powdered food supplemented with 1% w/w *β*‐GPA for 2 weeks, whereas the 6‐week treatment received 1% w/w *β*‐GPA diet for 6 weeks. Three grams of powdered mouse food per mouse were administered from a ceramic feeding dish daily, and mice from all groups consumed all of the food during each feeding. Mice were fed from the same dish for the extent of the treatment. All treatments were approved by the UNCW Institutional Animal Care and Use Committee.

### Muscle isolation

Mouse hind limb muscles were excised after CO_2_ euthanasia. The gastrocnemius muscle and the extensor digitorum longus (EDL), both of which are composed of predominantly fast‐twitch fibers, were isolated and excised from the hind limb. The gastrocnemius is relatively large, making it amenable for extraction and NMR analysis. The EDL, in contrast, is relatively small in cross section, making it useful for microscopic analysis since the entire muscle cross section can be readily evaluated (Luedeke et al. 2004; Overton et al. 2009). Immediately following excision, gastrocnemius muscle samples were freeze–clamped with metal tongs cooled in liquid nitrogen and then weighed. EDL muscles were immediately placed in a 4% paraformaldehyde fixative (16 mL of 20% paraformaldehyde and 84 mL of Sorensen's phosphate‐buffered saline [PBS]) and stored until used for microscopy.

### Perchloric acid extraction

Freeze‐clamped gastrocnemius muscle samples were transferred with nine volumes of 7% perchloric acid solution containing 1 mmol/L EDTA to a 50‐mm diameter agate mortar (Fisher Scientific) cooled in liquid nitrogen. The frozen muscle sample with perchloric acid solution was then manually pulverized into a fine powder using an agate pestle. The homogenate was centrifuged at 10,000*g* for 15 min at 4°C. The supernatant was neutralized with the appropriate volume of 3 mol/L potassium bicarbonate in 50 mmol/L PIPES, stored on ice for 10 min, and centrifuged at 10,000*g* for 15 min at 4°C. The supernatant high‐energy phosphate (HEP) molecules were immediately analyzed using ^31^P‐nuclear magnetic resonance (NMR) spectroscopy, which is a means of analyzing samples rapidly and thus limiting HEP breakdown.

### NMR spectroscopy

Gastrocnemius supernatants were transferred to 5‐mm NMR tubes and ^31^P‐NMR spectra were collected at a frequency of 162 MHz on a Bruker 400 MHz DMX Spectrometer. Two thousand four hundred scans were collected for each spectrum using a 45° excitation pulse with a 0.6 sec relaxation delay, resulting in a total acquisition time of 30 min. The relative peak areas of *α*‐, *β*‐, and *γ*‐ATP, PCr, P_i_, and P‐GPA were integrated using Xwin‐NMR software, and relative amounts of each compound were determined from the relative peak area.

### Microscopy

EDL muscles were rinsed in a 5% sucrose solution twice for 15 min, and refrigerated overnight in a 30% sucrose solution. At the midpoint of each muscle, approximately 10 transverse cross sections were cut at a thickness of 30 *μ*mol/L at −19°C on a Reichert‐Jung Cryocut 1800 cryostat. Sections were rinsed with Sorensen's PBS and stained with *Griffonia simplicifolia* lectin (GSL) for two hours (30 *μ*L GSL in 2970 *μ*L Sorensen's PBS) to label capillary endothelia. GSL was conjugated with an Alexa Fluor 488 fluorescent marker. Slides were then rinsed with Sorensen's PBS for an additional 15 min, followed by 30 min staining with wheat germ agglutinin (WGA) (8 *μ*L WGA in 992 *μ*L of Sorenson's PBS) conjugated with Alexa Fluor 594. WGA is used to stain the connective tissue around muscle fibers, and therefore outlines the fiber periphery. Following WGA staining, the samples were rinsed with Sorensen's PBS for 15 min. Slides were viewed on an Olympus FluoView 1000 laser scanning confocal microscope and images were collected in z‐stacks consisting of seven slices, 1 *μ*m thick with 20× magnification.

Fiber cross‐sectional area (FCSA), number of capillaries around a fiber (CAF), and capillary density (CD) were analyzed using Image Pro Plus Software and Adobe Photoshop version 7.0. A square lattice counting grid with 50 *μ*m by 50 *μ*m squares was randomly positioned over an image. Muscle fibers were selected for analysis using a systematic random sampling procedure (Howard and Reed, 1998) where fibers at every sixth grid intersection were evaluated. Approximately 100 fibers were analyzed in the EDL muscle per mouse. The muscle fibers were outlined on Adobe Photoshop and then the mean diameter and cross‐sectional area were calculated in Image Pro Plus. CAF was measured by a manual count of the number of capillaries touching each respective fiber. Capillary density was obtained by dividing the CAF by the muscle fiber cross‐sectional area for each individual fiber. Since there is an effect of sex on muscle mass (see Results), only female mice were evaluated for these measurements, and there were five mice analyzed for both the control and 6‐week *β*‐GPA‐treated groups.

### Measurement of basal metabolic rate

BMR was measured during the daytime (quiescent phase in mice) following the protocol of (Speakman and McQueenie 1996). BMR measurements began at least 3 h into the light phase of the 12‐h light/dark cycle and measurements were conducted before daily feeding. Feeding on the day prior to BMR measurements was conducted during the early afternoon. At 16 weeks of age, mice were weighed and transferred to an enclosed 2‐L respirometry chamber. The chamber was housed in an incubator maintained at the thermoneutral temperature for mice of 29°C (Gordon 1993). The incubator was equipped with a light source to ensure the animal was quiescent throughout the experiment. The respirometry chamber was continuously flushed with an air mixture that was rapidly equilibrated by a small fan at one end of the chamber. The flow rate of air was maintained with a Sierra Instruments Smart‐Trak digital mass flow controller at 50 mL/sec. Air was drawn through the chamber by a dual channel Applied Electrochemistry R‐2 flow controller. Air samples from the chamber and control were passed through columns of Drierite and Ascarite to remove water and CO_2_ and then through an Applied Electrochemistry dual channel S‐3A/II differential oxygen analyzer. Oxygen consumption was recorded for a period of 3 h and BMR was calculated as the mean value of the lowest oxygen consumption rate that was maintained at 1% variance for 4 min. Steady‐state *V*O_2_ measurements were determined using the following equation:

$$VO_{2}=V\left({\text{FiO}}_{2}\right)/1-{\text{FIO}}_{2}$$

V, flow of CO_2_ free dry air, FiO_2_, fractional concentration of O_2_ in the incurrent air and FeO_2_, fractional concentration of O_2_ in the excurrent air (Bartholomew et al. 1981; Kolb et al. 2010).

### Normoxic and hypoxic exercise tolerance model

Normoxic exercise tolerance (NET) and hypoxic exercise tolerance (HET) were measured as previously described (Luedeke et al. 2004; Overton et al. 2009). NET and HET are defined as the duration of treadmill exercise until exhaustion under either normoxic or hypoxic conditions. Mice were weighed prior to exercise and then placed into either a normoxic (21% O_2_) or a hypoxic compartment (10.5% O_2_) containing a treadmill inclined to 15° with an electrical grid at the base that produced a 0.15 mA scrambled current when two or more bars were touched simultaneously. The mice were allowed a 10 min acclimation period inside the compartment prior to the onset of treadmill exercise. The treadmill exercise was carried out at a speed of 40 cm/sec until exhaustion that was defined as the point when the mouse spent 4 consecutive seconds on the electrical grid. Upon exhaustion, the treadmill and electrical grid were turned off and the mouse was allowed to recover overnight before tissue collection.

### Statistical analyses

Shapiro–Wilk tests were used to assess normality of data, and Brown–Forsythe tests were used to assess homogeneity of variances. Most of the group data were distributed normally, and since analysis of variance (ANOVA) is robust to nonnormal distributions, we used two‐way ANOVA in cases where variance was homogeneous. We tested for significant effects of sex, treatment, and sex‐treatment interactions on body mass, muscle mass, relative [HEP], mass‐specific BMR, NET, and HET. Where significant effects were detected, Student's *t*‐tests were used to make post hoc pairwise comparisons. In cases where variance was not homogeneous, a Kruskal–Wallis (KW) test was used to identify significant effects of treatment and a Mann–Whitney *U*‐test was used for post hoc pairwise comparisons, as well as to test for sex differences. BMR was the only variable that was correlated with body mass, so analysis of covariance (ANCOVA) was used to analyze body mass‐corrected BMR. We tested for differences in mean fiber size and capillarity between the control and 6‐week *β*‐GPA groups using t‐tests. Results were considered significant if *P* < 0.05. All analyses were conducted using JMP statistical software.

## Results

### High‐energy phosphate compounds

**Figure 1:**
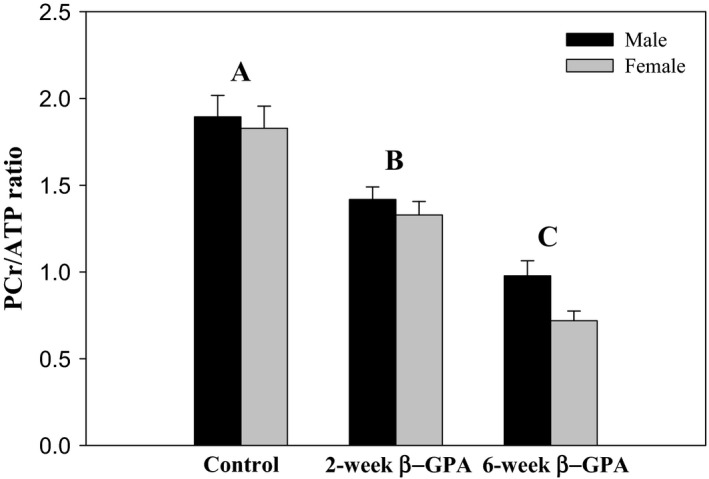
*β*‐GPA treatment reduces PCr/ATP. ANOVA detected a significant effect of *β*‐GPA treatment on the PCr/ATP ratio, but there was no effect of sex or a sex‐treatment interaction. Pairwise differences among treatments are indicated by different capital letters. *n *≥* *27 for each group (see Table 1).

NMR analysis yielded relative HEP concentrations for the gastrocnemius muscle, which are presented in Table 1. The variance for relative [PCr] and [P‐GPA] were not homogeneous, so a Kruskal–Wallis test was applied, but all other data were analyzed with 2‐way ANOVA. There was a significant effect of treatment on relative [PCr] [*χ* ^2^(2,220) = 136.05, *P *<* *0.0001), [ATP] [*F*(2,220) = 6.39, *P *<* *0.0020], [P_i_] [*F*(2,220) = 36.87, *P *<* *0.0001], and [P‐GPA] [*χ* ^2^(1,156) = 97.67, *P *<* *0.0001) (only the 2‐ and 6‐week *β*‐GPA treatments were considered for P‐GPA since this compound is absent in the controls). There were no significant effects of sex or a sex‐treatment interaction on relative concentrations of HEP molecules. Pairwise comparisons revealed that relative [PCr] and [P_i_] were higher in the control mice than in the 2‐ or 6‐week *β*‐GPA‐treated mice, relative [ATP] was higher in the control mice than in the 6‐week‐treated mice, and relative [P‐GPA] was higher in the 6‐week‐treated mice than in the 2‐week‐treated mice. The changes above led to a significant effect of treatment on the PCr/ATP ratio [*F*(2,220) = 55.19, *P *<* *0.0001], and pairwise comparisons revealed that the PCr/ATP ratio was significantly higher in control mice than in the 2‐ or 6‐week *β*‐GPA‐treated mice (Fig. 1).

**Table 1:** Relative concentrations of HEP metabolites in gastrocnemius muscle from control and *β*‐GPA‐treated mice

	Male			Female		
	Control	2‐week *β*‐GPA	6‐week *β*‐GPA	Control	2‐week *β*‐GPA	6‐week *β*‐GPA

PCr	0.241 ± 0.008^A^	0.173 ± 0.006^B^	0.105 ± 0.007^C^	0.224 ± 0.010^A^	0.166 ± 0.006^B^	0.087 ± 0.007^C^
*β*‐ATP	0.133 ± 0.004^A^	0.1316 ± 0.003^A^	0.110 ± 0.004^B^	0.138 ± 0.004^A^	0.133 ± 0.004^A^	0.117 ± 0.005^B^
P‐GPA	0	0.109 ± 0.007^A^	0.231 ± 0.009^B^	0	0.112 ± 0.005^A^	0.247 ± 0.011^B^
P_i_	0.144 ± 0.006^A^	0.089 ± 0.007^B^	0.101 ± 0.007^B^	0.140 ± 0.009^A^	0.093 ± 0.005^B^	0.078 ± 0.007^B^
*n*	27	35	42	37	44	35

Relative concentrations of high‐energy phosphates from ^31^P‐NMR experiments for each sex and treatment. There was a significant treatment effect in all measurements, and pairwise differences among treatments are indicated by different capital letters. The zero data for P‐GPA controls were not included in the analysis (see Results). There were no significant effects of sex or a sex‐treatment interaction. Data are means ± SEM.

### Body and muscle mass

**Figure 2:**
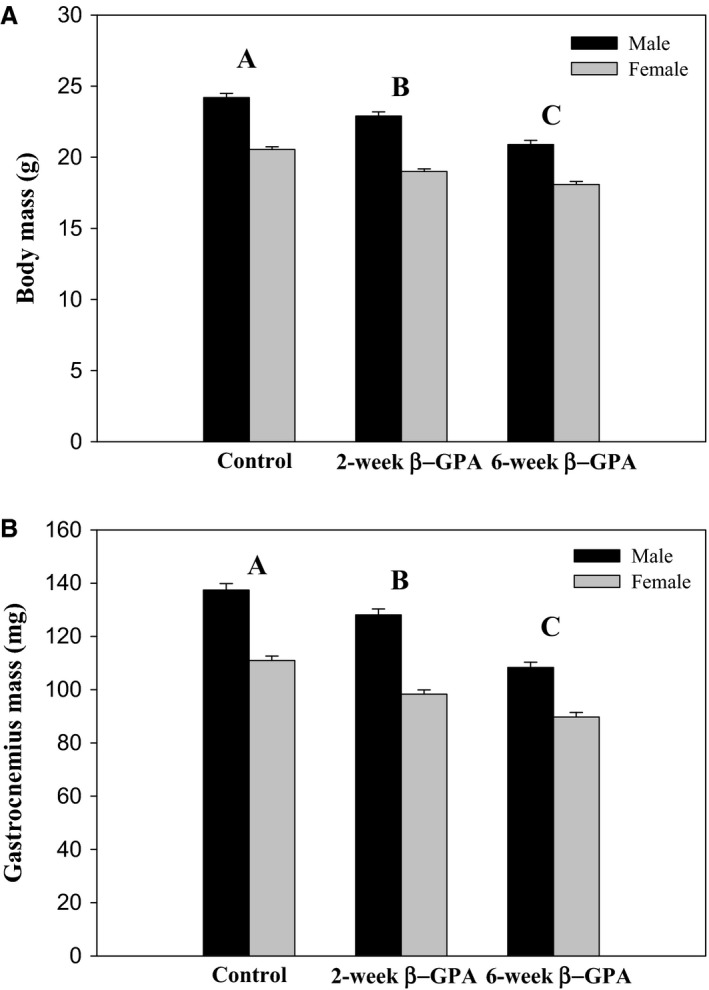
*β*‐GPA treatment decreases body mass and muscle mass. ANOVA detected a significant effect of treatment on body mass (A) and gastrocnemius mass (B) (pairwise differences among treatments are indicated by different capital letters). A significant effect of sex was also detected in body mass and muscle mass (not indicated on graph), where the males had a larger mass than females, but there was no sex‐treatment interaction. *n *≥* *31 for all experiments.

There was a significant effect of sex [*F*(1,242) = 305.73, *P *<* *0.0001] and treatment [*F*(2,242) = 69.7357, *P *<* *0.0001], but no sex‐treatment interaction, on body mass (Fig. 2A). Body mass was greater in males than in females and *β*‐GPA treatment led to a reduction in body mass in both sexes. There was also a significant effect of sex [*F*(1,231) = 246.17, *P *<* *0.0001], treatment [*F*(2,231) = 83.49, *P *<* *0.0001], and a sex‐treatment interaction [*F*(2,231) = 4.66, *P *=* *0.0104] on gastrocnemius mass (Fig. 2B). The gastrocnemius mass was greater in males than in females and *β*‐GPA‐treated mice had lower muscle mass than control mice.

### Muscle fiber size and capillarity

**Figure 3:**
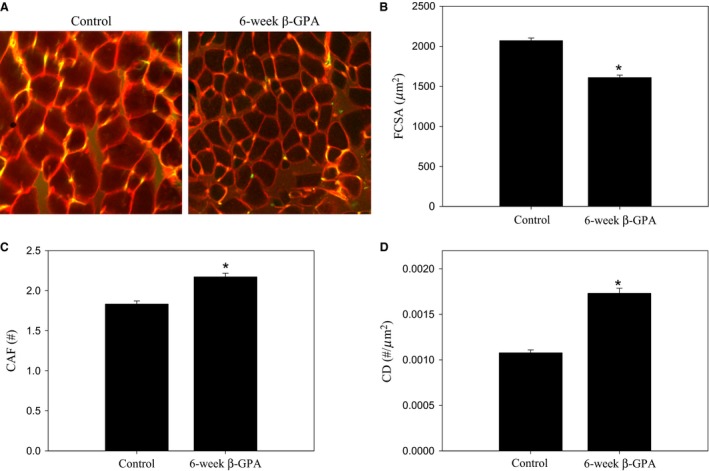
*β*‐GPA treatment reduces muscle fiber size and increases capillarity. Representative images showing extensor digitorum longus fibers stained red with wheat germ agglutinin and capillaries stained yellow with *Griffonia simplicifolia* lectin (A). *T‐tests revealed that following 6 weeks of *β*‐GPA treatment fiber cross‐sectional area (FSCA) was lower (B), whereas (CAF) a fiber capillaries around a fiber (CAF) (C) and capillary density (CD) (D) were higher. *n *=* *628 fibers measured in controls and *n *=* *527 fibers measured in *β*‐GPA‐treated mice.

Representative images from cross sections of the EDL can be seen in Fig. 3a. Following 6 weeks of *β*‐GPA treatment, FCSA was lower than in the controls (*t*‐test, *P *<* *0.0001) (Fig. 3B), whereas capillarity was higher than in controls, indicated by a greater CAF (*t*‐test, *P *<* *0.0001) (Fig. 3C) and CD (*t*‐test, *P *<* *0.0001) (Fig. 3D).

### Basal metabolic rate

**Figure 4:**
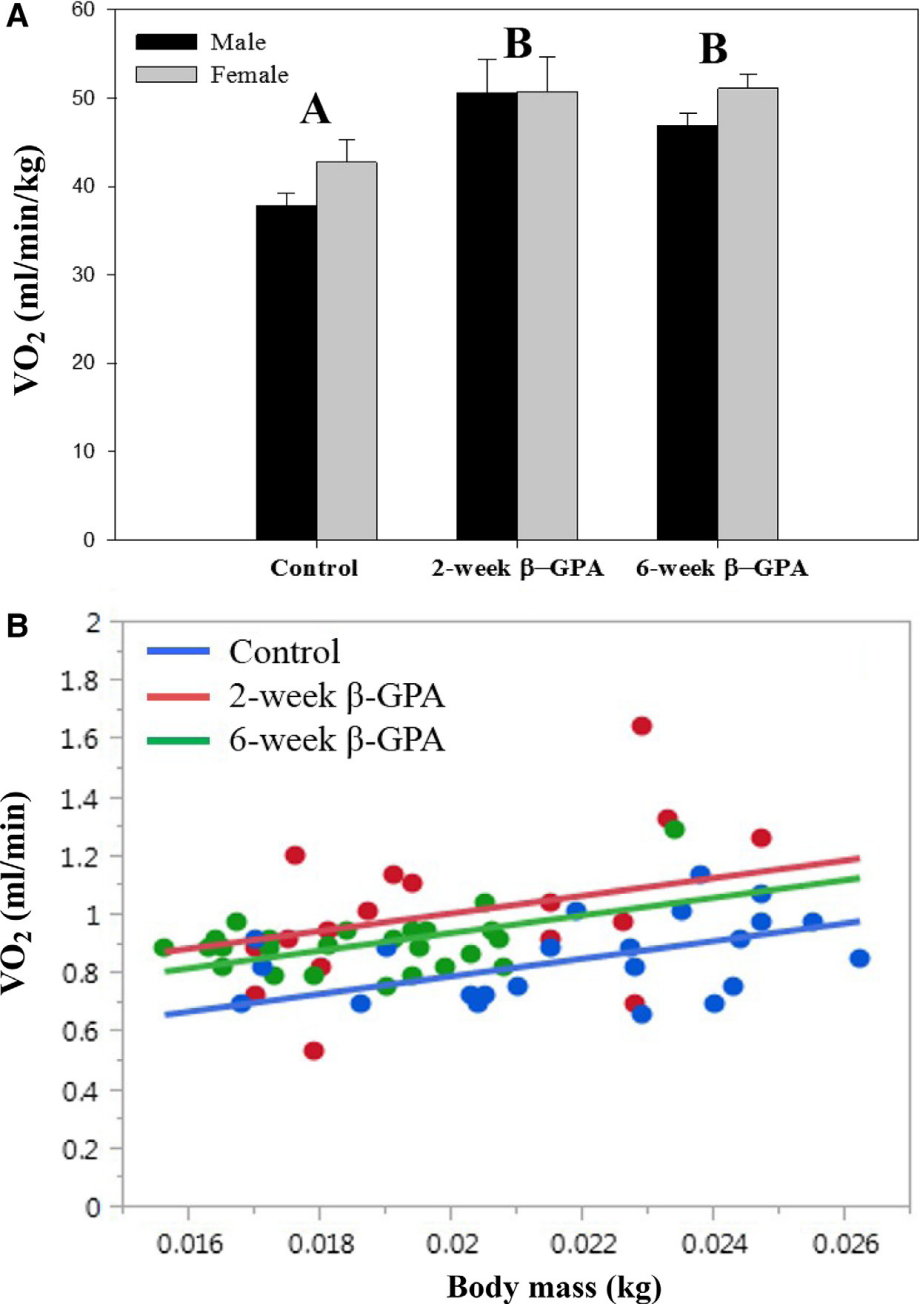
*β*‐GPA treatment increases basal metabolic rate. ANOVA detected a significant effect of treatment on mass‐specific BMR (A), and pairwise differences among treatments are indicated by different capital letters. There was no effect of sex or a sex‐treatment interaction. ANCOVA revealed that when compared at a common body mass, the treatment effect on BMR was still significant, and pairwise comparisons demonstrated that the 2‐ and 6‐week treatments were significantly greater than the controls (see results). *n *≥* *7 for each group in (A) *n *≥* *17 for each group in (B).

There was a significant effect of treatment on mass‐specific BMR [*F*(2,64) = 10.28, *P *=* *0.0001], but no significant effect of sex or a sex‐treatment interaction. Pairwise comparisons revealed that mass‐specific BMR was greater in both 2‐ and 6‐week *β*‐GPA fed mice than control mice (Fig. 4A). Two‐week *β*‐GPA‐treated mice had a 28% greater mass‐specific BMR than those of the controls and 6‐week *β*‐GPA‐treated mice had a 24% greater mass‐specific BMR than those of the controls. Since BMR was positively correlated with body mass, ANCOVA was used to evaluate the effect of the *β*‐GPA treatment at a common body mass (males and females were pooled as there was no significant effect of sex). The slopes of BMR on body mass were not significantly different among treatments, so the treatment‐body mass interaction term was not included in the analysis. ANCOVA revealed that there was a significant effect of treatment on BMR using body mass as a covariate [*F*(2,61) = 8.88, *P *<* *0.0004]. Pairwise comparisons showed that both the 2‐ and 6‐week *β*‐GPA‐treated mice had a higher BMR than did the control group (Fig. 4B).

### Normoxic and hypoxic exercise tolerance

**Figure 5:**
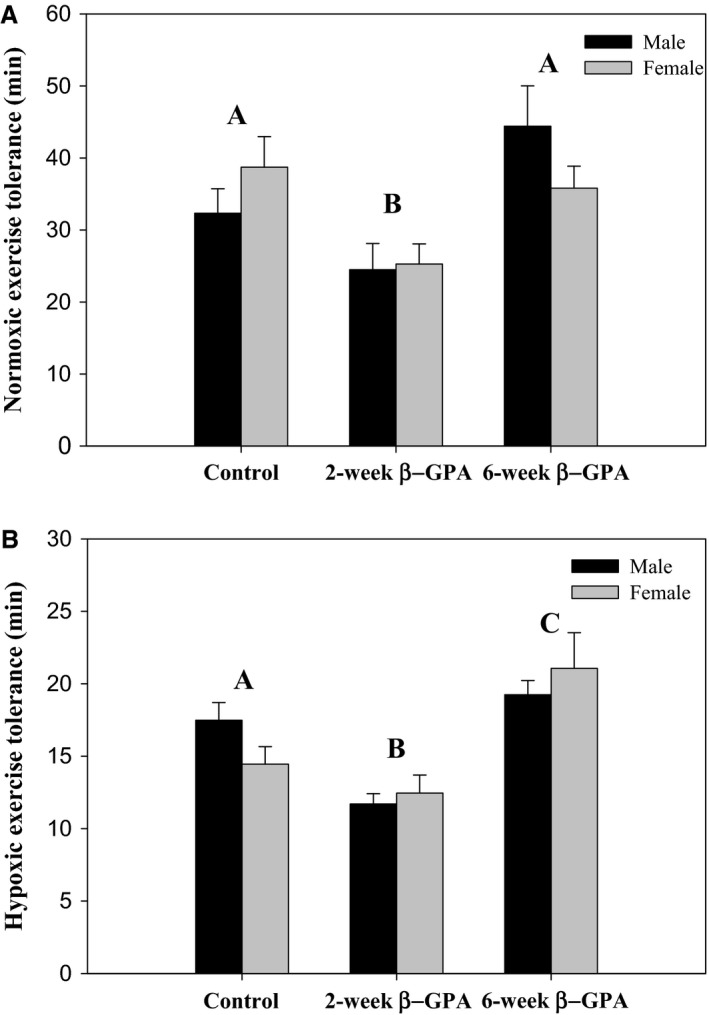
6‐week *β*‐GPA treatment enhances hypoxic exercise tolerance. ANOVA revealed a significant treatment effect on both normoxic (A) and hypoxic exercise (B). Pairwise differences among treatments are indicated by different capital letters. There was no effect of sex or a sex‐treatment interaction in either NET or HET. *n *≥* *18 for each group.

There was a significant effect of treatment on NET [*F*(2,119) = 7.97, *P *=* *0.0006] (Fig. 5A) and HET [*F*(2,122) = 16.98, *P *<* *0.0001] (Fig. 5B), but no effects of sex or sex‐treatment interactions. Pairwise comparisons revealed that the 2‐week *β*‐GPA‐treated mice had a lower NET and HET than control, whereas the 6‐week *β*‐GPA‐treated mice had a significantly higher HET, but not NET, than the control.

## Discussion

This study demonstrated that both 2‐ and 6‐week *β*‐GPA treatments resulted in a lower muscle PCr/ATP ratio, decreased body mass, muscle mass, and fiber size, and increased capillarity and BMR. Most of these responses are similar to those following hypoxia acclimation, so it is not surprising that mice treated with *β*‐GPA for 6 weeks had an increased exercise tolerance under hypoxic conditions.

### *β*‐GPA and environmental hypoxia lead to similar energetic changes

Previous studies have shown that in skeletal muscle *β*‐GPA leads to a reduction in the PCr/ATP ratio (Shoubridge et al. 1985; Ren et al. 1993; Freyssenet et al. 1995; Bergeron et al. 2001; Reznick et al. 2007; Williams et al. 2009), fiber type switching toward more aerobic, slow twitch fiber types (Shoubridge et al. 1985; Freyssenet et al. 1995; Ecochard et al. 2004; Chaturvedi et al. 2009), and improved contractile economy (Moerland and Kushmerick 1994). This study demonstrated a reduction in PCr/ATP after *β*‐GPA feeding similar to prior studies that is likely associated with a shift toward more aerobic fiber types, which in untreated muscle have a lower PCr/ATP ratio than anaerobic, fast‐twitch fibers (Kushmerick et al. 1992).

Acclimation to hypoxia causes energetic shifts in muscle comparable to *β*‐GPA treatment. The PCr/ATP ratio in cardiac muscle is lower in hikers following an excursion to high altitude (Holloway et al. 2011), as well as in native highlander Sherpas (Hochachka et al. 1996). We have previously shown that a reduced PCr/ATP is also an acclimation response in skeletal muscle of mice exposed to hypobaric hypoxia for 8 weeks, and that this is associated with an increase in ADP and AMP, but a reduced inorganic phosphate (P_i_) (Overton et al. 2009). The reduction in P_i_ preserved the Δ*G* of ATP hydrolysis, which would otherwise have been compromised by the changes in the concentrations of PCr, ADP, and AMP. Thus, a reduced PCr/ATP appears to be a muscle tissue level response to environmental hypoxia in mammals. However, in our hypoxic mouse model, the reduction in PCr/ATP following acclimation to hypoxia is not associated with fiber type shifts (Luedeke et al. 2004), and the lack of fiber type changes with hypoxia acclimation is consistent with studies in rat (Ishihara et al. 1995), guinea pig (Jackson et al. 1987) and humans (Green et al. 1989). These findings suggest that the changes in HEPs associated with hypoxia acclimation play a metabolic role that is independent of fiber type.

We have previously found that hypoxia acclimation in mice also leads to increases in resting metabolic rate and maximal metabolic rate under hypoxia (unpubl. data), as well as substantially increased hypoxic exercise tolerance (McCall and Frierson 1997; Luedeke et al. 2004; Overton et al. 2009). Similarly, Lui et al. (2015) found that maximal O₂ consumption during exercise in hypoxia increased after hypoxia acclimation and was consistently greater in highland deer mice compared to lowland deer mice, and Suzuki (2016) showed that intermittent hypoxia with training led to greater hypoxic endurance in mice than did training alone. Taken together, the fiber type‐independent reduction in PCr/ATP during environmental hypoxia, and the associated increases in oxygen consumption and exercise capacity make it reasonable to test the hypothesis that a *β*‐GPA induced reduction in PCr/ATP leads to an increase in BMR and hypoxic exercise tolerance.

### *β*‐GPA elevates basal metabolic rate

This study found a significant increase in BMR in both males and females after *β*‐GPA treatment. While the influence of *β*‐GPA treatment on BMR has not been previously studied, resting metabolic rate (RMR) has been found to increase in rodents in response to *β*‐GPA (Tanaka et al. 1997) or the AMP analog, AICAR (Narkar et al. 2008). While the mechanisms by which both RMR and BMR are elevated are likely to be the same, RMR includes the influence of activity, thermoregulation, nutrient absorption, and the circadian cycle. In contrast, BMR is a measure of the minimal maintenance costs required to sustain the organism, and it is independent of potential behavioral changes associated with *β*‐GPA treatment (Speakman and McQueenie 1996). It is likely that at least some of the increase in BMR following *β*‐GPA treatment is mediated by AMPK, which is activated by elevated AMP concentration and is a key cellular energy sensor. Perturbations in HEP compounds induced by *β*‐GPA supplementation increase AMPK activity (Bergeron et al. 2001; Williams et al. 2009; Baumgarner et al. 2015), and AMPK activation results in mitochondrial biogenesis (Zong et al. 2002; Jager et al. 2007; Karagounis and Hawley 2009; Canto et al. 2010), increased capillary density (Thomas et al. 2014), increased oxidative enzymes and increased glucose uptake (Oudman et al. 2013). Therefore, the capacity to produce ATP is likely elevated following AMPK activation by *β*‐GPA.

Since skeletal muscle is metabolically plastic and represents 30–40% of BMR in mammals (Field et al. 1939, 1993; Zurlo et al. 1990), enhanced muscle metabolism may account for much of the increase in BMR reported in this study, although changes in metabolism of other tissues are also likely to be important. The large increase in BMR seen following the 2‐week *β*‐GPA treatment probably results from extensive tissue remodeling associated with this acute energetic stress (Shoubridge et al. 1985; Freyssenet et al. 1995; Ecochard et al. 2004; Chaturvedi et al. 2009). However, the less extreme increase in BMR seen in the 6‐week *β*‐GPA treatment likely reflects a new steady‐state BMR where mitochondrial density in muscle and other tissues is elevated. This view is supported by the smaller fiber size and increased capillarity we observed in the EDL following *β*‐GPA treatment, which is consistent with a shift toward more aerobic fiber types. While CD can increase due to the reduction in fiber size, the increase in CAF is indicative of the formation of new capillaries and a higher muscle oxygen demand. There was also a 12–13% reduction in body mass after 6 weeks of *β*‐GPA treatment. Previous studies have found both small (Shoubridge et al. 1985; Freyssenet et al. 1995) and moderate (Tanaka et al. 1997; Robinson and Loiselle 2002) decreases in rodent body mass with *β*‐GPA administration, which may be explained by the higher BMR and tissue remodeling that occurs in treated mice.

BMR also has been shown to correlate with maximal metabolic rate (MMR) (Bennett and Ruben 1979; Bozinovic 1992; Hinds and Rice‐Warner 1992; Rezende et al. 2004), and MMR is often positively related to exercise capacity (Bassett and Howley 1997). Prior studies have found that in rodents a higher BMR is associated with a higher MMR among different species (Bozinovic 1992; Rezende et al. 2004) and within species (Hayes 1989; Chappell and Bachman 1995), consistent with the aerobic capacity model (Bennett and Ruben 1979). MMR is determined by the energy needs of the cells that are active during maximal work, which is determined by the quantity of oxidative enzymes and the capillary density (Weibel and Hoppeler 2005). Thus, an increase in the mitochondrial density and capillary density should result in an increase in MMR, BMR, and exercise tolerance. In addition, it has been shown that mice artificially selected for high BMR had increased voluntary activity (Gebczynski and Konarzewski 2009), whereas mice with impaired AMPK function had decreased voluntary activity (Thomson et al. 2007), suggesting a direct link between cellular energy state, BMR, and voluntary activity that is likely partly mediated by AMPK.

### *β*‐GPA increases hypoxic exercise tolerance

This study found a significant decrease in both NET and HET following a 2‐week *β*‐GPA treatment, despite the large increase in BMR. The decrease in exercise tolerance is likely due to acute tissue remodeling that transiently compromises muscle function. After the 6‐week *β*‐GPA treatment, however, there was a significant increase in HET, but not NET, although the pattern for normoxic endurance was the same as that for hypoxic endurance. Acclimation to hypoxia also yields a reduced PCr/ATP ratio in both mouse skeletal muscle and human cardiac muscle that is associated with enhanced exercise endurance under hypoxic conditions (Hochachka et al. 1996; Wu et al. 2005; Overton et al. 2009). HEPs are altered by *β*‐GPA in tissues such as cardiac muscle, smooth muscle, kidney, and brain, but the perturbations to energetic state and the functional effects are less dramatic than in skeletal muscle (reviewed in Oudman et al. 2013). Thus, the energetic changes induced by *β*‐GPA likely instill a range of responses that lead to improved hypoxic endurance, but a proximate cause appears to be the well‐established alteration in HEPs in muscle that mirrors the responses of muscle to exercise training and hypoxia acclimation.

The fact that this and other studies have found a link between HEPs in muscle and hypoxia tolerance may have broader implications. For instance, the apparent reduction in cellular energy state that accompanies tissue hypoxia in certain disease states may represent an acclimation response to hypoxia that aids cellular function under these conditions, rather than an energy imbalance per se.

## Conflict of Interests

The authors have no competing interests or conflict of interest regarding this manuscript.
